# Rosiglitazone disrupts pancreatic ductal adenocarcinoma progression by activating the tumor suppressor ESE3/EHF

**DOI:** 10.20892/j.issn.2095-3941.2022.0299

**Published:** 2022-07-21

**Authors:** Hongquan Wang, Shuang Wu, Yan Wang, Bo Tang

**Affiliations:** 1 Department of Pancreatic Cancer, Tianjin Medical University Cancer Institute & Hospital, National Clinical Research Center for Cancer, Key Laboratory of Cancer Prevention and Therapy, Tianjin, Tianjin’s Clinical Research Center for Cancer, Tianjin 300060, China

Pancreatic ductal adenocarcinoma (PDAC), the most common histologic type of pancreatic cancer, is among the most lethal cancers, and is associated with poor prognosis because of its inherent chemoresistant nature and metastatic capacity^1^. Extensive evidence has shown that cancer stem cells (CSCs), a subpopulation of highly plastic “stem”-like cells within the PDAC, mediate this chemoresistantce and metastatic capacity^2^. CSCs have unique metabolic, invasive, and chemoresistance properties that enable them to continually self-renew and escape standard chemotherapeutic elimination in PDAC, thus often rendering treatments ineffective^3,4^.

Ongoing investigations have indicated that CSCs are regulated by both cell-intrinsic signaling pathways and cell-extrinsic factors derived from stromal cells^5,6^. Pancreatic stellate cells (PSCs), the major cell types in the PDAC stroma, show abundant desmoplasia, which promotes cancer cell aggressiveness and resistance to anti-cancer drugs. These important cell-extrinsic factors secrete prostemness cytokines, thereby forming the CSC niche and participating in active crosstalk with cancer cells within the tumor microenvironment^7–9^. Consequently, targeted attenuation of PSC activation is a promising therapeutic strategy to inhibit tumor-stromal interactions with pancreatic cancer cells^7,10^. However, current anti-CSC therapeutic strategies focus mainly on targeting cell-intrinsic stemness-associated genes, which are shared by CSCs and normal stem cells, thus resulting in adverse effects. Consequently, an urgent need exists to understand the complexity of stromal expansion and dysfunctional tumor immunity to develop effective therapies for PDAC.

**Figure 1:**
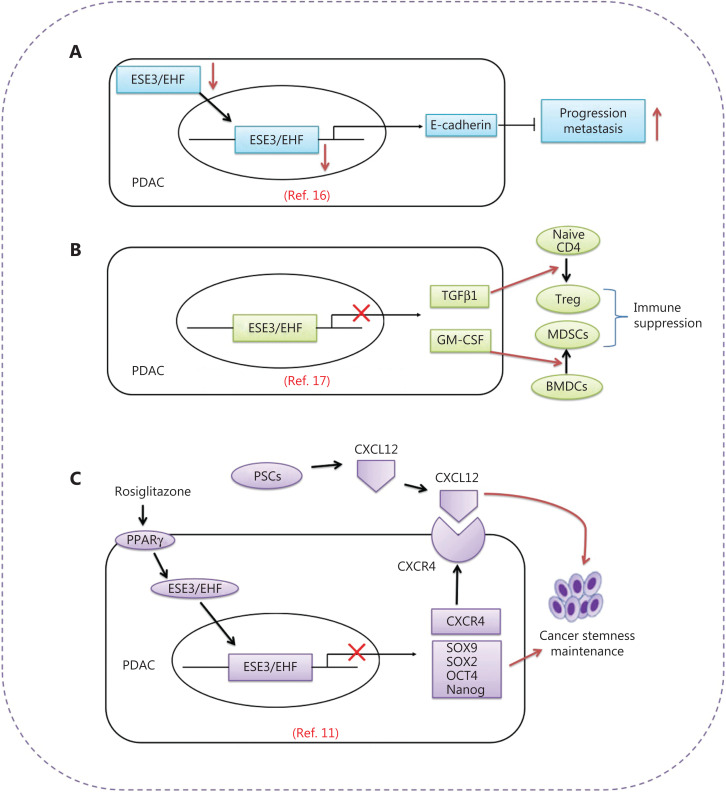
Schematic depiction of the roles of the tumor suppressor ESE3/EHF in PDAC. (A) ESE3/EHF inhibits PDAC metastasis through direct transcriptional upregulation of E-cadherin expression in PDAC^16^. (B) ESE3/EHF decreases tumor-infiltrating T reg cells and MDSCs through transcriptional suppression of the expression of TGFβ1 and GM-CSF^17^. (C) ESE3/EHF is a tumor cell-intrinsic and tumor cell-extrinsic master transcription factor that disrupts the crosstalk between CSCs and their PSC-derived CSC-supportive niche by negatively regulating tumor CXCR4. Targeting ESE3/EHF by rosiglitazone provides an alternative strategy to induce PDAC regression^11^.

A study published in *Gut* by Zhou et al.^11^ demonstrates that epithelium-specific E26 transformation-specific (ETS) factor family member 3 or ESE3/E26 transformation-specific homologous factor (ESE3/EHF) functions as a tumor cell-intrinsic and cell-extrinsic master transcription factor that disrupts the crosstalk between CSCs and the PSC-derived CSC-supportive niche by negatively regulating the CXCL12 receptor CXCR4 in tumors. Targeting of ESE3/EHF by rosiglitazone thus provides an alternative strategy to induce PDAC regression^11^ (**Figure 1**).

ESE3/EHF, a member of the Ets family of transcription factors, is a tumor suppressor transcription factor^12,13^ that is crucial in differentiation and development programs for many epithelial tissues, and plays an important role in cancer development^14^. ESE3/EHF is frequently down-regulated in several malignancies, and is involved in carcinogenesis and progression^15^. Setting the stage for these new findings, Zhao et al.^16^ first suggested a role of ESE3/EHF in PDAC development and progression (**Figure 1A**). In their early work, the authors showed that the expression of ESE3/EHF is markedly diminished in PDAC, and is closely correlated with lymph node metastasis, vessel invasion, and attenuated relapse-free and overall survival in patients. Downregulation of ESE3/EHF has been found to promote PDAC cell motility and invasiveness, together with metastasis, in an orthotopic mouse model. Mechanistic studies have indicated that ESE3/EHF inhibits PDAC metastasis through direct transcriptional upregulation of E-cadherin expression in PDAC cell lines and an orthotopic mouse model. Moreover, in human PDAC specimens, ESE3/EHF has been found to inhibit PDAC metastasis through direct transcriptional upregulation of E-cadherin expression^16^. Extending these findings, the authors have demonstrated that ESE3/EHF has novel roles in edition of pancreatic cancer immune microenvironment and prediction of the efficacy of anti-PD1 therapy. Loss of tumoral ESE3/EHF induces accumulation of regulatory T (T reg) cells and myeloid-derived suppressor cells (MDSCs), and decreases the number of tumor-infiltrating CD8^+^ T cells. Mechanistic studies have demonstrated that ESE3/EHF deficiency induces the conversion and expansion of T reg cells and MDSCs through inhibition of tumor TGFβ1 and GM-CSF secretion, respectively. EHF suppresses the transcription of TGFβ1 and GM-CSF by directly binding their promoters. Mice bearing ESE3/EHF overexpressing tumors show a significantly better response to anti-PD1 therapy than mice bearing control tumors. These findings have indicated the immunosuppressive mechanism of ESE3/EHF deficiency in PDAC, thus highlighting that ESE3/EHF overexpression may improve PDAC checkpoint immunotherapy^17^ (**Figure 1B**).

Using LSL-Kras^G12D/+^mice, a LSL-Trp53^R172H/+^ and Pdx1-Cre (KPC) mouse model, and samples from patients with PDAC, Zhou et al.^11^ have provided the first evidence that ESE3/EHF acts as a driver of malignant tumor progression, and suppresses cancer stemness through both cancer cell-intrinsic and cancer cell-extrinsic mechanisms. These findings provide a compelling rationale for therapeutic targeting of ESE3/EHF. In their studies, the authors have demonstrated that ESE3/EHF represses the expression of SOX9, SOX2, OCT4, and Nanog in the cell-intrinsic pathway. Simultaneously, ESE3/EHF decreases the sensitivity of PDACs to stimuli from the PSC-derived CSC-supportive niche by negatively regulating tumor CXCR4 expression in the cell-extrinsic pathway^11^. Rosiglitazone could conceivably be used to target pancreatic stem cells and the crosstalk between CSCs and their niche by upregulating ESE3/EHF^11^. Rosiglitazone, a specific PPAR-γ agonist that improves glycemic control and insulin sensitivity in patients with diabetes by selectively activating PPAR-γ, inhibits PDAC stemness and suppresses sensitivity to stemness-promoting stimuli by upregulating ESE3/EHF expression. Additionally, rosiglitazone has been found to sensitize PDAC to gemcitabine therapy in a KPC mouse model. Together, these findings elucidate how ESE3/EHF mediates the crosstalk between pancreatic cancer and its PSC-associated stemness-supporting niche. Developing therapies targeting this crosstalk through modulation of ESE3/EHF by rosiglitazone may be feasible and would have great therapeutic potential (**Figure 1C**).
